# Preservative Monitoring of a Greek Woman with Hydrops Fetalis due to Parvovirus B19 Infection

**DOI:** 10.1155/2017/4389321

**Published:** 2017-07-12

**Authors:** Zacharias Fasoulakis, Panagiotis Antsaklis, Emmanuel N. Kontomanolis

**Affiliations:** ^1^Department of Obstetrics & Gynecology, Democritus University of Thrace, Alexandroupolis, Greece; ^2^1st Department of Obstetrics & Gynaecology, Kapodistrian University of Athens, Athens, Greece

## Abstract

Primate erythroparvovirus 1 (parvovirus B19) is a member of the* Erythrovirus* genus of the Parvoviridae family and it is one of the few members of the family known to be pathogenic in human. B19 infection is common and widespread with the virus being associated with numerous rheumatologic and haematologic manifestations. More specifically, maternal infection with parvovirus B19 during pregnancy can cause severe anemia which may lead to nonimmune hydrops or fetal demise, as a result of fetal erythroid progenitor cells infection with shortened half-life of erythrocytes. We present a rare case reported in the Greek population, of subclinical transient reticulocytopenia due to B19 parvovirus infection, in an asymptomatic pregnant woman, without medical history of hemoglobinopathy, and with the presence of hydrops fetalis during the third trimester of her pregnancy.

## 1. Introduction

Infections of primate erythroparvovirus 1 (also known as parvovirus B19) are responsible for erythema infectiosum, polyarthropathy syndromes, and transient aplastic crisis in patients with underlying hemolytic disorder in both adults and children [1, 2]. Among adults, more than 50% have contracted the virus with a lifelong maintenance of immunity. Maternal infection with parvovirus B19 during pregnancy can cause severe anemia which might lead to nonimmune fetal hydrops and fetal death [3–5].

Nonimmune hydrops fetalis, a rare condition with an unknown etiology in 20–50% of cases, occurs mostly between 11 and 23 weeks of gestation after maternal B19 infection [6, 7]. Recent studies report that hydrops development, after infection is confirmed, is about 1–1,6% while fatality rate is almost 50% and 18% with and without intrauterine transfusion, respectively [8–12].

Despite the high fatality rate, there are only a few data for the Greek population considering B19 infection during pregnancy and there are no cases reported about fetal hydrops caused by B19 virus. We present a case report of a 35-year-old pregnant woman of Greek origin, being at the 21st week of gestation, with the presence of viral DNA, transient aplastic crisis, and hydrops fetalis due to parvovirus B19 infection that without further therapy led to a safe pregnancy result.

## 2. Case Presentation

A 35-year-old, Greek, pregnant woman was examined in our Obstetrics Clinic on the basis of a regular gestation surveillance at the 21st week of gestation. The woman had no medical history of thalassemia and she was pregnant for the second time. Her first delivery was performed with cesarean section. Her obstetric ultrasound revealed 21-week gestational age, normal placenta location (grade 0), and fetal heart beat from 140 to 150 bpm. The patient did not mention any unusual symptoms. No vaginal bleeding was noted. After approximately 3 weeks, her first child presented signs of erythema infectiosum on the head, torso, and upper extremities. The pediatrician estimated parvovirus B19 infection and recommended serum laboratory data of the woman in order to diagnose possible transmission by her first child. Being 24 weeks' pregnant, she was again presented to our Obstetrics Clinic. Apart from a mild fatigue, pulse rate of 92 beats/min, and respiratory rate of 18 breaths/min, no major clinical manifestations were reported. Her laboratory data showed values of haematocrit estimated at 31,6%, hemoglobin 10.8 g/dl, and red blood cell count 3.74 M/*μ*l.

Appropriate volumes of whole blood were drawn from the patient. The serum fractions were allowed to clot at room temperature prior to centrifugation. The serum samples had been stored at −20°C until tested for B19 IgM antibody, IgG antibody, and B19 DNA. A commercial kit ELISA (RecomWell; Mikrogen GmbH, Neuried, Germany) based on parvovirus B19 recombined capsid proteins was used for the detection of anti-B19 IgM and IgG antibodies in serum.

**Figure 1:**
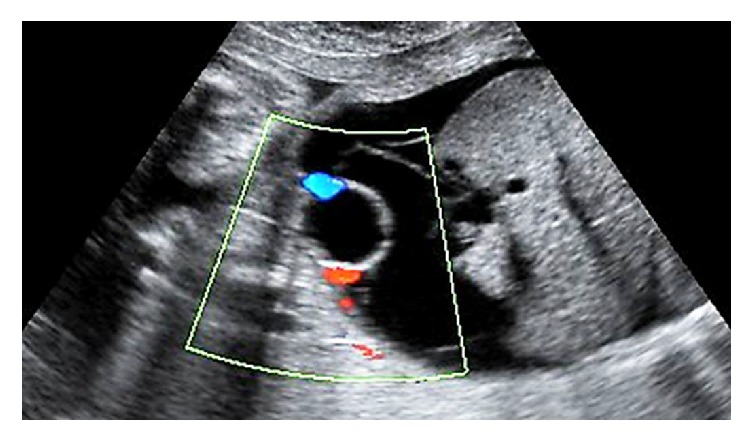
Ascites in the peritoneal cavity with the two umbilical arteries encircling the urinary bladder.

Parvovirus serology (anti-IgM/IgG) was determined using ELISA. Consecutive IgM-IgG tests confirmed the diagnosis. The first detection, 4 weeks before the cesarean section, revealed IgG: 40 U/mL and IgM: 8.4 U/mL (<0,9 negative) and the second detection after 3 weeks revealed IgG: 35 U/mL and IgM: 2,8 U/mL. The patient's blood was tested once more 2 weeks afterwards and the results were IgG: 35 U/mL and IgM: 2,8 U/mL that indicated a recent infection. The woman was under close surveillance with serum tests and ultrasound-Doppler imaging. The condition of the fetus was normal and stable. A couple of weeks later, ultrasound revealed the presence of ascites within the peritoneal cavity as an indication of hydrops fetalis (Figure 1).

For the viral detection, a parvovirus genotype 1–3-specific TaqMan real-time PCR assay was also included, for the assessment of B19 DNA level. Commercially available kit was used for the isolation and the extraction of DNA (Qiagen GmbH, Hilden, Germany) following the manufacturer's instructions. For quantification the international standard 99/800 was used (NIBSC, Potters Bar, UK). In all previous samples for set time points the B19V-DNA was detected (1.0 × 10^3^ to 1.7 × 10^12^ geq/mL).

Bone marrow biopsy was not performed and direct and indirect antiglobulin tests were not examined because haemolytic anemia was not included in the differential diagnosis.

The condition of the gestation was well-controlled, without any unusual signs and symptoms, when three months later oligohydramnios was presented, due to rupture of the fetal membranes, and a cesarean section was performed resulting in a normal looking healthy baby boy (Apgar score 8 at 1′ and 9 at 5′, body weight 3108 gr). Cord blood of the newborn was tested for parvovirus B19 to prove vertical transmission. After delivery, her reticulocytopenia was absent.

## 3. Discussion

Parvovirus B19 is a single-stranded DNA virus of the family Parvoviridae and genus* Erythrovirus* and the first human virus of this family to be discovered in human blood samples [2]. Correlation of parvovirus and aplastic crisis has been confirmed [13–15]. Moreover, B19 infection has been recognized as an etiologic factor of erythema infectiosum in haematologically normal persons, while cases of nonimmune hydrops fetalis were reported by Anderson and Hurwitz in 1988, when a pregnant woman was diagnosed with B19 infection [16, 17]. The incubation period usually ranges from 4 to 14 days, the rash usually occurs 2-3 weeks after initial infection, and patients are most contagious few days before the rash [18]. Failure of differentiation from proerythroblast into later stage erythroid precursors leads to transient aplastic crisis in patients with shortened lifespan of erythrocytes because of an underlying haemolytic problem, such as spherocytosis, sickle cell anemia, autoimmune haemolytic anemia, thalassemia, and G6PD deficiency [2]. B19 infection may present pancytopenia; however, its role as an etiology of true aplastic anemia is ambiguous [18].

In order to evaluate the risks of perinatal outcome (before confirmation of maternal infection) for pregnant women of Greek origin, MEDLINE was used to detect reports of acute Parvovirus B19 infections during pregnancy which were addressed to Greek hospitals. Exindari et al. reported in 2011 the epidemiological and clinical characteristics for B19 infections in northern Greece while Daniilidis et al. presented in 2014 a case-study control of 206 pregnant women infected by B19 virus during the 2005–2009 period. However, in contrast to international literature, there are no reports considering the main manifestation caused by B19 virus infection during pregnancy in the Greek population [19, 20].

Nonimmune hydrops fetalis is rare (1 in 3,000) with unknown etiology in many cases (20%–50%) and may occur when a nonimmune woman is infected by B19 parvovirus, usually in the first 20 weeks of pregnancy and can result in fetal death in 2–6% of cases [6, 7, 18]. Many women of childbearing age are susceptible to infection and the seroconversion rate is estimated at 1.5% per year [10]. From the infected fetuses, in the first half of pregnancy, 85% develop hydrops within 10 weeks, but severe anemia after 21 weeks is not observed [5].

A case of maternal severe transient aplastic crisis, on the background of shortened lifespan of erythrocytes, was recently reported due to B19 infection during pregnancy [21]. In addition, Rajput et al. reported a case of severe aplastic anemia in a previously healthy adult female because of acute parvovirus B19 infection [22].

Our patient presented transient aplastic crisis without known history of haemoglobinopathy, a case rarely reported. Furthermore, we observed indication of hydrops fetalis in the second half of gestation. Erythrocyte transfusion, corticosteroids, IVIG, or intrauterine transfusion were not administered, even though spontaneous resolution of hydrops fetalis is only 34% [23]. Weekly fetal ultrasound examination and adequate surveillance led to a safe delivery of a healthy baby without any complications. Imaging studies and blood tests of a 1-month follow-up were normal. Since the infected children living at home represent the major infectious source, a vaccine could probably be the most suitable precautionary measure [8].
